# Supplementary material for: CHRM3 (rs2165870) gene polymorphism is related to postoperative vomiting in female patients undergoing laparoscopic surgery. Prospective observational study
Source: PLoS One. 2024 Aug 20;19(8):e0309136. doi: 10.1371/journal.pone.0309136 (PMC11335095; doi:10.1371/journal.pone.0309136)
Supplement: S1 Protocol — (DOCX) [file pone.0309136.s003.docx]

**The correlation study between genes polymorphisms with analgesia effects and postoperative vomiting in female patients undergoing laparoscopic surgery**

**Research protocol (V1.0)**

The content of this study is included in the overall project "Postoperative individualized PCIA analgesia based on the ADRA2A, OPRM and COMT genes".

**Project summary**

**The rationale:** Postoperative nausea and vomiting (PONV) are the most common side effects experienced after anesthesia, and the relationship between gene polymorphisms and the occurrence of PONV has attracted increasing attention in recent years. Previous research indicated that CHRM3 rs2165870 and the KCNB2 rs349358 SNP have significant impacts on the incidence of PONV, particularly in Caucasians

**Objectives:** This study aimed to explore the relationship between the CHRM3 rs2165870 polymorphism and postoperative vomiting incidence in female patients who underwent laparoscopic surgery.

**Methods:** The CHRM3 rs2165870 and KCNB2 rs349358 polymorphisms were genotyped using MassARRAY SNP typing technology. Demographics and preoperative laboratory examinations of all patients were recorded. Postoperative analgesic-related information, incidence of postoperative nausea and vomiting, and other adverse events were followed up and recorded for analysis.

**Populations:** Female patients receiving elective laparoscopic surgery with patient-controlled intravenous analgesia using dexmedetomidine and sufentanil were prospectively enrolled.

**Time frame:** 1) Before August 2022, the research team will discuss, formulate the implementation plan, implement the details, determine the study design and obtain ethical approval. 2) Participants were enrolled from August 2022 to June 2023, and data were collected. 3) June 2023 thereafter: Statistical analysis and conclusion of the results.

**Expected outcomes:** There is a correlation between a certain genes polymorphisms and the occurrence of PONV in female patients who underwent laparoscopic gynecological surgery followed by sufentanil for postoperative pain relief.

**General information**

- Date:2022.08- 2023.07
- This study was supported by the Natural Science Foundation of Chongqing of China (No. cstc2021jcyj-msxmX0763) and the National Key Clinical Speciality Construction Project (Obstetrics and Gynecology).
- PI: Jin Yu, Doctor-in-charge, Department of Anesthesiology.
- Institution: Department of Anesthesiology. Chongqing Health Center for Women and Children/Women and Children’s Hospital of Chongqing Medical University.

**Rationale & background information**

1. **Why did this study take place?**

Single nucleotide polymorphisms (SNPs) are the main cause of interindividual differences in drug response, leading to the same therapeutic drug being highly effective for some populations while having little or reduced efficacy for others. The effectiveness and toxicity of drugs mainly depend on the pharmacokinetic processes of the drugs in the body, including absorption, distribution, metabolism, and excretion, any alterations in which can affect the safety of the drug. SNPs are variations in single nucleotides in the human genome, with approximately 90% of human gene DNA sequences having variations in this form. There are numerous genetic markers formed by SNP variations, and the number of polymorphisms is high. Therefore, SNPs have become third-generation genetic markers, and various phenotypic differences between different human populations, susceptibility to drugs or diseases, etc., may be related to SNPs. Genetic background differences, namely, SNPs, are important factors in generating individual differences in the pharmacokinetics of analgesic and sedative drugs. It can be inferred that this genetic polymorphism is also a key factor in producing individual differences in postoperative analgesia.

Laparoscopic surgery, characterized by minimal trauma and rapid postoperative recovery, is increasingly widely used in clinical practice. This technique has high application value, especially in gynecological surgery. However, patients still experience varying degrees of pain after surgery, which affects their rapid postoperative recovery and reduces their medical comfort. The combination of the highly selective α2-adrenergic receptor agonist dexmedetomidine and sufentanil for patient-controlled intravenous analgesia (PCIA) after laparoscopic gynecological surgery provides precise analgesia and sedation effects, improved postoperative cardiovascular stability, high patient satisfaction, and a low incidence of respiratory depression at appropriate drug doses. However, the analgesic effect of fixed drug concentrations varies among different patients.

Sufentanil is a fentanyl analog that primarily acts on μ-opioid receptors. It is widely used as an intravenous, subarachnoid, epidural anesthetic and postoperative analgesic with strong efficacy and high lipid solubility. The μ-opioid receptor, encoded by the OPRM1 gene (OPRM1), serves as the primary target for sufentanil. OPRM1 (rs1799971) has been demonstrated to result in the substitution of asparagine with aspartate at position 40, leading to elevated pain scores and increased morphine usage. Within the CYP3 enzyme family, CYP3A4 is a pivotal hepatic microsomal enzyme that is crucial for metabolizing 45-60% of therapeutic medications. It plays a significant role in the processing of opiates and benzodiazepines. Parturients carrying CT heterozygotes and TT homozygotes of CYP450 3A4*1G (rs2242480) required a lower dosage of sufentanil during epidural labor analgesia. Dexmedetomidine is a highly selective α2 adrenergic receptor (α2‑AR) agonist that is widely used for postoperative analgesia. Individuals carrying the G allele of ADRA2A (C1291G) tend to require higher levels of sedation when dexmedetomidine is administered than do those with the C allele. Specifically, different variants of ADRA2A, such as rs11195418, rs1800544, rs553668, and rs10885122, have shown significant associations with diverse sympathetic responses.

The CHRM3 rs2165870 polymorphism has been found to be associated with the efficacy of ondansetron in preventing PONV in the Chinese Han population. A meta-analysis also indicated that CHRM3 rs2165870 and the KCNB2 rs349358 SNP appear to have significant impacts on the incidence of PONV, particularly in Caucasians.

Exploration of the influence of these genetic variants on the effectiveness of pain relief from opioids and other pain-related SNP loci could pave the way for tailored postoperative opioid management strategies based on individual genetic profiles. This approach holds the promise of mitigating the chances of insufficient pain control, as well as minimizing the occurrence of nausea, vomiting and respiratory depression. To achieve this objective, a series of SNP sites were detected in this study for analysis. These sites include opioid receptor, metabolism-related, pain sensitivity-related and dexmedetomidine receptor polymorphisms. The aim of this study was to identify distinct sites associated with postoperative analgesia in patients undergoing laparoscopic surgery. This endeavor lays the groundwork for refining the approach to individual postoperative analgesia administration.

In the case of female patients undergoing laparoscopy, the administration of opioids for postoperative analgesia, along with the influence of gender itself, may undeniably contribute to an increased risk of PONV. Thus, it is necessary to assess the impact of the CHRM3 rs2165870 and KCNB2 rs349358 polymorphisms on the occurrence of PONV among female patients who underwent elective laparoscopic surgery and received patient-controlled intravenous analgesia.

1. **Risks and adverse reactions of participants in this study?**

In this observational study, SNP testing was free of charge and did not incur additional medical expenses for patients. Blood collection while awake may cause slight discomfort to patients. Patients participating in this study will receive detailed preoperative pain assessments and close follow-up and observation of postoperative pain management. Dedicated nurses will follow up on postoperative pain and recovery progress.

**Study goals and objectives**

(1) To explore the distribution of common gene mutation sites (ADRA2A (C1291G), OprM A118G and COMT Val158Met) and other pain-related genes (CHRM3 rs2165870 and KCNB2 rs349358) in women undergoing laparoscopic surgery in this area;

(2) To analyze the effects of genetic factors, i.e., SNPs, on pain sensitivity and the postoperative analgesic effect of PCIA in female patients undergoing laparoscopic surgery;

(3) To provide a theoretical basis for individualized postoperative PCIA in patients with different genotypes and guide individualized postoperative analgesic medication.

**Study design**

**Inclusion criteria**:

① Female, aged ≥ 18 years and < 60 years, ASA I-III;

② Plans to perform gynecological laparoscopic surgery under general anesthesia and require postoperative PCIA;

③ Patients who had normal communication skills, could correctly use the postoperative patient-controlled intravenous analgesia pump, and could cooperate to evaluate the pain intensity;

④ No history of narcotic drug allergy;

⑤ Patients who were able to understand the contents of the informed consent form and make judgments independently, agreed to participate in this study and signed the informed consent form.

**Exclusion criteria**:

① Mental illness and cognitive impairment;

② Allergy to known analgesic and sedative drugs;

③ Patients with a history of chronic pain and a long history of using analgesic and sedative drugs;

④ Patients with peripheral neuropathy symptoms and abnormal liver and kidney function;

⑤ Failure to implement as planned, incomplete data records, and affecting the judgment of pain intensity;

⑥ Patient refused.

**Exclusion criteria not included in the statistical analysis**:

① Conversion to open surgery during laparoscopic surgery;

② Patients with a surgical duration exceeding 3 hours;

③ Patients who experienced serious complications during surgery, such as major bleeding, requiring admission to the ICU.

According to the inclusion and exclusion criteria, surgical patients were selected, informed consent was obtained, and informed consent forms were signed before the patients entered the study. Venous blood was drawn for SNP genotyping. After laparoscopic surgery, intravenous PCIA was administered according to the established analgesia protocol. During the postoperative analgesia period, patients' tolerance to pain was closely monitored, and detailed records of drug dosage, effectiveness, relevant complications, adverse reactions, etc., were kept. Patient grouping was based on the results of genetic testing when the data were ready for statistical analysis.

### Methodology

All patients underwent a standardized protocol of general intravenous anesthesia with tracheal intubation, which was initiated using a controlled infusion of remifentanil (with a target plasma concentration of 5 ng/ml), propofol (with a target plasma concentration of 3.5 µg/ml), a single intravenous injection of sufentanil (0.25 μg/kg) and rocuronium (0.6 mg/kg). Anesthesia was continuously maintained using propofol and remifentanil in TCI mode, with additional doses of rocuronium administered as needed.

For PCIA, a combination of dexmedetomidine (100 µg), sufentanil (75 µg), dexamethasone (10 mg) and normal saline was used for a total volume of 100 ml. The analgesic pump utilized was the PCA-100C type, a disposable adjustable infusion pump manufactured by Zhejiang Chen He Medical Equipment Co., Ltd., China. The infusion was administered continuously at a rate of 2 ml/h, and a lockout time of 15 minutes was set. Patients were advised to self-administer a dose of 0.5 ml by pressing the control button.

**Measurements**

**Pain Relief Effectiveness Indicators**

(1) Pain relief effectiveness indicators: Pain intensity was assessed using visual analog scale (VAS) scores, which were recorded at 2 h, 4 h, 8 h, 12 h, and 24 h postoperatively.

(2) Ramsay sedation scale: 1 Agitated; 2 Awake, calm, and cooperative; 3 Drowsy, responsive to commands; 4 Light sleep, easily awakened; 5 Asleep, sluggish response to call; 6 Deep sleep, unresponsive to call.

(3) Total analgesic drug dosage: The amount of sufentanil used within 24 hours postoperatively was recorded.

(4) The number of rescue analgesia instances was recorded.

(5) Number of self-administered analgesic presses.

(6) Patient self-reported satisfaction.

(7) Sleep time on the night after surgery.

Safety indicators:

Adverse event occurrence:

(1) Hypotension (SBP <90/60 mmHg),

(2) Respiratory depression (SpO2 <90%),

(3) Bradycardia (HR <50 beats/min),

(4) Nausea and vomiting

(5) Dizziness, drowsiness,

(6) Urinary retention and incidence of other adverse events.

**Therapeutic efficacy and safety assessment criteria**

No special intervention was applied in this study, except for the additional collection of blood for SNP testing. All other procedures were conducted according to routine diagnosis and treatment.

**Follow-up**

If the subject has any questions related to this study, please contact the attending physician or researcher Meng Cai at 17302304865. If the subject has any questions related to the rights and interests of the patient, please contact the Medical Ethics Committee of Chongqing Maternal and Child Health Hospital at 023-63316835.

**Data management and statistical analysis**

This was a prospective study, and the sample size was estimated using GPower 3.1.9.2 software. Based on previous literature (Li Zhi. Research on the Application of Dexmedetomidine in Obstetric Epidural Anesthesia and Postoperative Analgesia and Related Gene Polymorphisms [D]. Postoperative 12-hour VAS scores and Ramsay sedation scores were selected as the main indicators for calculation. Postoperative 12-hour VAS and Ramsay sedation scores for the ADRA2A C1291G (rs1800544) locus wild-type homozygous group (C1229C) and mutant homozygous group (G1229G) were input into GPower 3.1.9.2 software.

(1) Extracting literature data for postoperative 12-hour VAS scores revealed that the C1229C group had a score of 1.7±0.7, and the G1229G group had a score of 3.4±1.6. Calculations were conducted with a Type I error probability of α=0.05, a test power of 1-β=0.9, an effect size d=1.377, and a ratio of n2/n1=1. The results indicated that each group required 10 cases, as shown in the figure below.


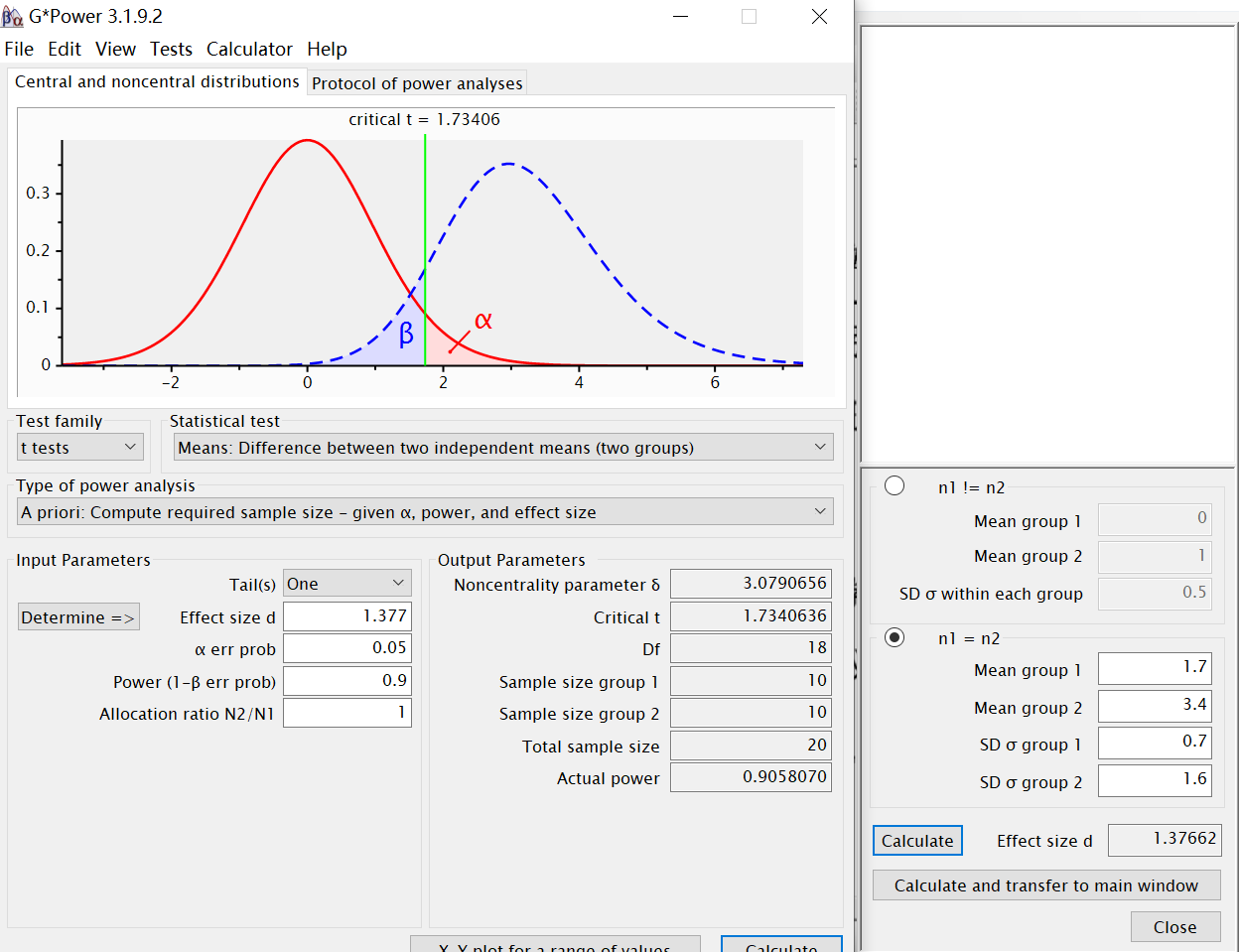


(2) The Ramsay sedation score at 12 hours postoperatively was 2.4±0.6 in group C1229C and 1.2±0.4 in group G1229G. According to the first type of error probability a=0.05, test power 1-β=0.9, allowable error effect size d=1.7231, and n2/n1=1 for calculation, the results indicate that each group requires 7 cases, as shown in the following figure.


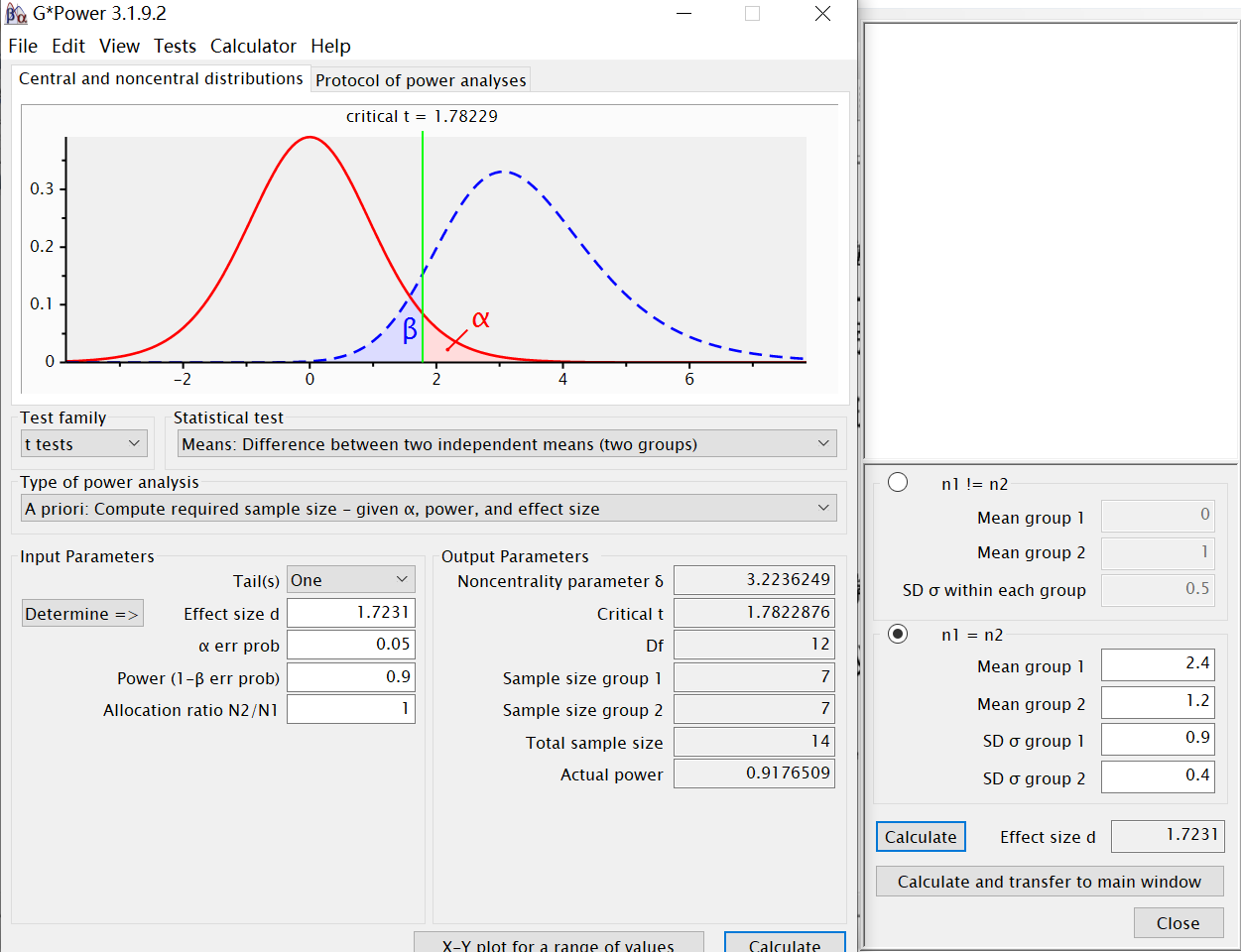


The distribution frequency of the sites in the literature was extracted. According to the genetic polymorphism detection report of ADRA2AC1291G, the frequencies measured for each group were as follows: C1291C (CC group 38 patients, 39.58%), C1291G (CG group 46 patients, 47.92%), and G1291G (GG group 12 patients, 12.5%). Since the distribution of G1291G accounts for 12.5%, at least 10 cases of homozygous mutations are needed. Therefore, including a minimum of 80 patients in the statistical analysis is necessary. It is expected that there will be a failure rate of 15-20% in SNP extraction and the exclusion of cases, as well as an approximately 20% mutation rate deviation. Therefore, the total sample size should not be less than 134 cases.

(3) Because both female sex and laparoscopic surgery are risk factors for PONV, combined with data from the literature (PMID: 18633022, PMID: 29843509), the baseline incidence rate of PONV after such surgeries is estimated to be 30-50%. Based on the mutation proportion of CHRM3 (PMID: 18633022), it is expected that the proportions of the GG, GA, and AA genotypes are 45%, 45%, and 10%, respectively. G Power 3.1.9.2 software was used to determine the sample size before this study under the following conditions: α = 0.05, β = 0.2, power value = 0.8, and estimated odds ratio (OR) = 2.79. The total sample size required was 167.

(4) Statistical analysis

Statistical analysis was performed using SPSS 25.0 statistical software. Normally distributed quantitative data are expressed as the mean ± standard deviation (±s). The chi-square test was used to examine whether the distribution of alleles and genotypes conformed to Hardy-Weinberg equilibrium (HWE). One-way analysis of variance (ANOVA) was used for comparisons of quantitative data among multiple groups. Homogeneity of variance was first tested, and when homogeneity was met, pairwise comparisons between group means were conducted using the least significant difference (LSD) method. If the variance was not homogeneous, group comparisons were made using the Dunnett method. Analysis of covariance was used to compare pain sensitivity and analgesic consumption among different genotypic groups to control for other influencing factors, such as age and BMI. The occurrence rates of postoperative adverse reactions among the groups were compared using the chi-square test or Fisher's exact probability method. Postoperative visual analog scale (VAS) scores were compared among the groups using the rank sum test. A p value less than 0.05 was considered to indicate statistical significance.

**Quality assurance**

All members of the research team received unified training before the start of the experiment and were familiar with the experimental operation procedures and specifications. The original data are recorded in the CRF table for unified management in a true, accurate, complete and timely manner. All procedures were performed by the same experienced anesthesiologist. The observation and follow-up personnel did not know the specific groups of patients. The final data were analyzed and summarized by personnel who did not participate in the specific experiment.

**Results and publication policy**

The results will be disseminated in the scientific media or scientific journals. In addition, we consider dissemination to policy makers to be relevant.

**Subject Recruitment and Protection Measures**

(1) Selection criteria, recruitment plan, and procedures for participants' research:

Participants aged between 18 and 60 years, with no history of allergy to anesthesia drugs, are scheduled for gynecological laparoscopic surgery under general anesthesia with postoperative patient-controlled intravenous analgesia (PCIA). They should possess normal communication abilities, be able to correctly use postoperative patient-controlled intravenous analgesia pumps, and cooperate in evaluating pain. Those who meet these criteria will be recruited after screening by the researcher among scheduled surgery patients.

(2) Explanation of the informed consent process:

Participants will be thoroughly briefed on the research's objectives, basic content, procedures, methods, and duration, as outlined in the informed consent document. They will also be informed about the researcher's credentials, institutional qualifications, potential benefits and discomforts, and risks associated with the research. The study is deemed to carry no potential adverse risks to patient safety.

Participants will emphasize that participation is entirely voluntary, that they may withdraw from the trial at any time without explanation, and that participation will not affect their treatment process. Medical records will be kept confidential in the hospital and accessible to the researcher, supervisory authorities, and ethics committees. During the trial, participants can request information or consult the supervising physician if issues arise.

(3) Measures for protecting participant privacy and confidentiality of information:

Any public reports on the research results will not disclose personal identifying information such as names or hospital numbers. Every effort will be made within legal limits to safeguard the privacy of personal medical data.

(4) Fair compensation for research participants and whether it is free:

Participants will not receive any remuneration for participating in this study, nor will it increase their treatment expenses during hospitalization. The additional SNP testing required for the trial will be provided free of charge, and no fees will be charged. Other charges and examinations will be consistent with those for regular patients. Follow-up doctors will pay particular attention to participants' pain changes and indicators during the postoperative analgesia period.

(5) Plan for reporting adverse events:

This study is observational and prioritizes patient safety. Any adverse events related to the research resulting in harm to participants' bodies or functions will be promptly documented in adverse event reports and reported to the hospital's ethics committee.
